# Seasonality of food-related anaphylaxis admissions and associations with temperature and pollen levels

**DOI:** 10.1016/j.jaip.2020.07.032

**Published:** 2021-01

**Authors:** Holly C.Y. Lam, Paul J. Turner, Debbie Hemming, Deborah L. Jarvis

**Affiliations:** a National Heart and Lung Institute, Imperial College, London, United Kingdom; b MRC Centre for the Environment and Health, Imperial College London, London, United Kingdom; c Section of Inflammation, Repair, and Development, National Heart and Lung Institute, Imperial College London, London, United Kingdom; d Discipline of Paediatrics and Child Health, School of Medicine, University of Sydney, Sydney, NSW, Australia; e Met Office Hadley Centre, Exeter, United Kingdom

**Clinical Implications**

• This is the first quantification of seasonality of food-related anaphylaxis admissions. Risk of food-related anaphylaxis admissions was higher around June in England (22% higher vs January), especially among children younger than 15 years. Health care professionals should consider this in managing patients at risk of food-related anaphylaxis.

Food-related anaphylaxis may be associated with seasonal ambient exposures, such as temperature^1^ and pollen.^2^ To date, state-of-the-art statistical techniques for identifying seasonal trends and potential associations with ambient exposures have not been applied to the available data. Here, using time series analysis, we assess seasonality in food-related anaphylaxis hospitalizations in England, to determine whether there is any association with ambient temperature and pollen exposure.

We obtained monthly counts of food-related anaphylaxis hospital admissions (T78.0) in England, and central England mean monthly temperature and calculated average monthly pollen count for the period 2010 to 2018. Admission and temperature (but not pollen) data were also available for the period 1998 to 2010. We used Poisson generalized additive models for time series to examine seasonality of admissions and the association of admissions with monthly mean temperature and pollen (exposure in the same and the previous month) from the period 2010 to 2018 (see the Methods section in this article's Online Repository at www.jaci-inpractice.org). Seasonality of admission and the temperature-admission associations were also examined in the extended data from 1998 to 2018.

From 2010 to 2018, there were 15,405 hospital admissions for food-related anaphylaxis (25,903 from 1998 to 2018) (see Table E1 in this article's Online Repository at www.jaci-inpractice.org). There was a seasonal trend in admission (Figure 1), with a peak in June (relative risk [RR], 1.22; 95% CI, 1.13-1.32, compared with January). In children younger than 15 years, this peak was particularly marked (RR, 1.39; 95% CI, 1.26-1.54, compared with January), but it was less apparent in young adults (RR, 1.14; 95% CI, 1.05-1.25) and not present in adults older than 45 years (1.01; 95% CI, 0.95-1.07). This pattern was also seen when evaluating all available admissions data (1998-2018) (see Figure E1 in this article's Online Repository at www.jaci-inpractice.org).

**Figure 1:**
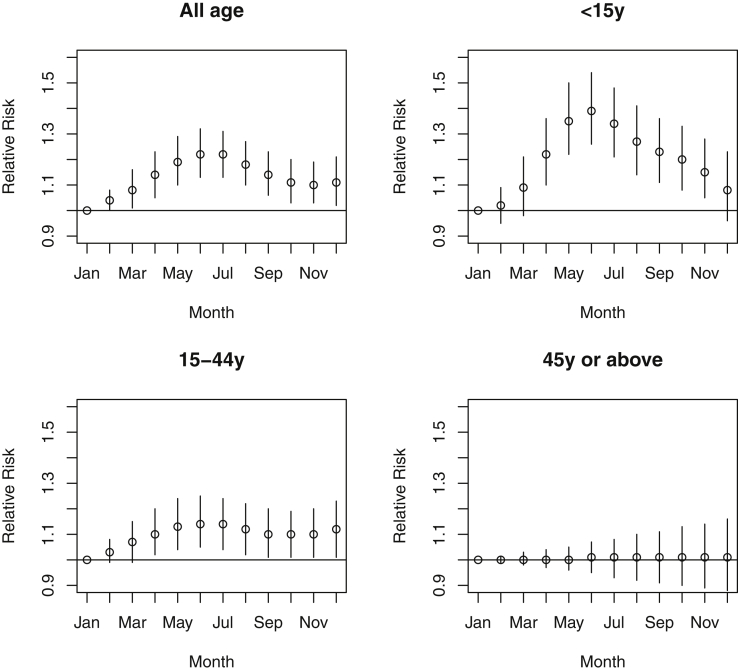
RR of food-related anaphylaxis admissions in each month (comparing to January) by age group, England, 2010-2018. Models were adjusted for yearly trend (y: year old).

We compared the admission risks between exposure levels at the 95th and 5th percentile. There was increased risk of admissions for *Ambrosia* (same month mean; RR, 1.16; 95% CI, 1.03-1.31) and *Quercus* (same month mean; RR, 1.12; 95% CI, 1.04-1.21) and, when a lag was considered, for *Fraxinus* (lagged 1 month mean; RR, 1.05; 95% CI, 1.01-1.10) (Figure 2). Unexpectedly we observed a significantly *decreased* risk for same month levels of *Fraxinus* (same month mean; RR, 0.94; 95% CI, 0.88-0.99), *Poaceae* (same month mean; RR, 0.86; 95% CI, 0.77-0.96), and *Urtica* (same month mean; RR, 0.85; 95% CI, 0.77-0.94). Of the 11 pollens we tested, *Betula, Fraxinus, Salix*, or *Ulmus* appeared to explain the seasonal peak of admissions in June with a delay effect, because the seasonal peak disappeared when lagged-1-month count of any of these 4 was included in the model.

**Figure 2:**
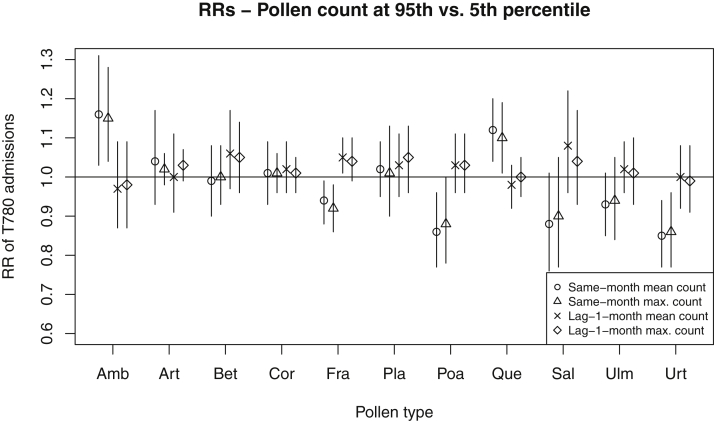
RR of food-related anaphylaxis admissions comparing pollen level at the 95th percentile and 5th percentile by pollen types, England, 2010-2018. Monthly mean and maximum (max.) pollen count in the same month and lagged 1 month are shown.

There was a nonsignificant (*P* > .05) positive association of admission with temperature (RR, 1.07; 95% CI, 0.99-1.16 per 10°C increase) from 2010 to 2018. In the full data set (1998-2018), this association reached statistical significance (RR, 1.10; 95% CI, 1.06-1.14 per 10°C increase). The seasonal peak in June of admissions was noted, even after accounting for temperature.

Our analysis shows seasonality in hospital admissions due to food-related anaphylaxis with a peak around June. This seasonality may be partly related to ingestion of seasonal food and, as suggested in our analysis, the variation in individual pollen levels. Compatible with this, 1 study in Sweden showed that the number of food-related anaphylaxis events among children allergic to pollens increased during the deciduous tree pollen season.^2^ A small study in Japan reported a peak of systemic allergic symptoms due to ingestion of soybean products during and after birch season in adults sensitized to both Bet v 1 and Gly m 4.^3^

Certain specific pollens, namely, *Fraxinus* (Ash), *Ambrosia* (Ragweed), and *Quercus* (Oak), were associated with higher risk of admissions. Research to date suggests that patients with pollen food allergy syndrome are at a lower risk of severe reactions to the causative allergen,^4^ so it is unlikely that cross-reactivity between food allergens and seasonal aeroallergens is the main driver of the seasonal peak observed. However, the above-mentioned report from Japan^3^ raises the possibility that sensitization to a pollen-homologous food protein component, with or without primary food sensitization, can lead to an increased risk of systemic reaction during the pollen season.

Alternatively, people who are sensitized to aeroallergens have a higher level of mast cell density in the airway,^5^ and nonspecific exposure to aeroallergen may increase their susceptibility to more severe respiratory symptoms (and therefore admission) on ingestion of food allergens.^5^ This may be particularly likely in patients with food allergy with asthma, who often report seasonal exacerbations during the pollen season. Such a phenomena would not involve cross-reactivity, and thus would not be limited to foods implicated in pollen food allergy syndrome. Higher levels of sensitization to pollens among the younger age groups or more recent cohorts^6^ might explain the stronger seasonality seen in these groups. Of note, the more frequent physical activities among children in spring-summer may also contribute to the summer peak in this age group.

We have shown that even low-level exposure to *Ambrosia* (ragweed) is associated with increased food-related anaphylaxis hospitalizations, which is of interest in light of likely increases in level of *Ambrosia* in England due to climate change.^7^

Unexpectedly, we found a negative association with a few genera/families (eg, *Fraxinus* and *Poaceae*). The underlying reasons are uncertain, but this may be related to an artifact related to the extreme lack of variation in some pollen levels from June onward (eg, *Fraxinus). Poaceae* includes pollen of all grass species. Admissions may have different associations with different grass species. A more detailed regional and species-specific study would be needed to explore this further.

Although both extreme hot and cold temperatures have been implicated as effect modifiers for food-dependent exercise-induced anaphylaxis,^1^^,^^8^ our work does not support strong associations of temperature with admissions. However, we have only considered monthly mean (rather than daily temperatures) and this negative finding should be interpreted cautiously. Current laboratory-based work suggests that higher environmental temperatures promote the production and delivery of immune cells to effector sites whereas low temperature tends to slow these processes.^9^ Using daily data with more personal characteristics of triggers may help in refining the temperature-food-related-anaphylaxis associations.

Our study is the first nationwide study of seasonal trends in food-related anaphylaxis and has used appropriate time series analysis to examine exposure-outcome associations accounting for the year-by-year increasing number of admissions. However, our hypothesis generating analyses has limitations including limited data relating to the causes (and potential miscoding) of food-related anaphylaxis admissions, the crude spatiotemporal scale of the analysis related to confidentiality of data, the potential inflation of type I error rate due to multiple testing, and not adjusting for other pollens or temperature due to inadequate power. In using a large national data set, we were unable to link specific patient episodes to patient-specific clinical data.

In summary, we report seasonality in recorded food-related anaphylaxis admissions in England, which may, in part, be related to pollen exposure. Overall risk of food-related anaphylaxis admission should be considered higher around June in England, and may indicate a pollen-driven process that is most relevant to a patient's risk profile; it is important for health care physicians caring for patients with food allergy to be aware of this. Analyses based on more refined spatiotemporal scales and with more clinical detail regarding ingestion of food that triggered the reaction may go some way to clarify these associations.

## Figures and Tables

**Figure E1:**
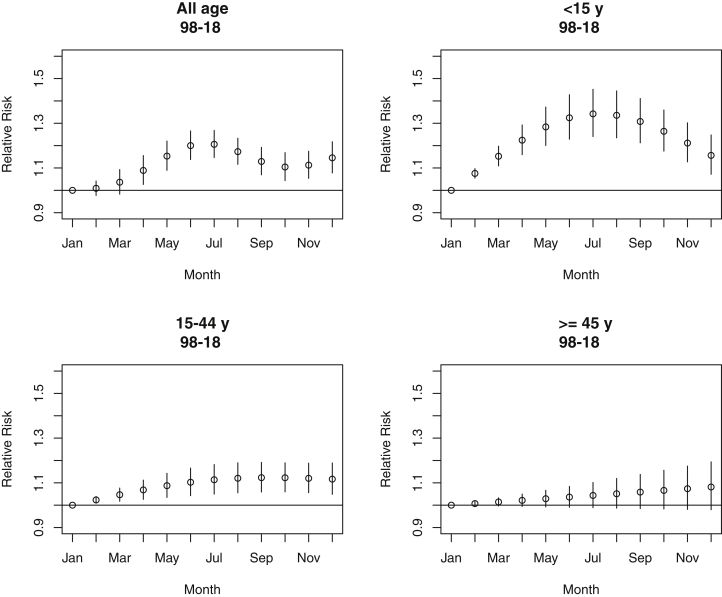
RR of food-related anaphylaxis admissions in each month (comparing to January) by age group, England, 1998-2018. Models were adjusted for yearly trend (y: year old).

**Table E1:** Age and sex distribution of food-related anaphylaxis (*ICD-10* code T780) admissions in England

Admission category	Number of admissions (%) in 2010-2018	Number of admissions (%) in 1998-2018
Total	15,405	25,903
Sex		
Female	7827 (50.80)	13248 (51.14)
Male	7578 (49.20)	12655 (48.86)
Age group (y)		
<15	5663 (36.76)	9425 (36.39)
15-44	7291 (47.33)	12014 (46.38)
45-64	2451(16.91)	4464 (17.23)

*ICD-10*, *International Classification of Diseases, Tenth Revision*.

## Online Repository

### Methods

#### Admissions data

We obtained monthly data from the Hospital Episodes Statistics database relating to hospital admissions for England in which food anaphylaxis (anaphylactic reaction due to food, T78.0) was the primary diagnostic code, for the calendar years 1998 to 2018. Hospital Episodes Statistics (coordinated through NHS Digital) collates all hospital admissions data in public hospitals in England

#### Pollen data

Pollen data were available from January 2010 to September 2018. Monitoring was conducted through the network of pollen monitoring stations run by the UK Met Office in collaboration with the National Pollen and Aerobiology Research Unit in the University of Worcester.^E1^ Pollen samples were collected daily or weekly during the pollen season (usually from late March to the beginning of September) using Burkard traps. Genera- or family-specific pollen counts were made by experts using microscopy.

Daily pollen count data (grains/m^3^/24 hours) were downloaded from the Medical & Environmental Data Mash-up Infrastructure database.^E2^ These included information from 10 stations in England covering 12 pollen genera/families: Trees—*Alnus* (alder), *Betula* (birch), *Corylus* (hazel), *Fraxinus* (ash), *Platanus* (plane), *Quercus* (oak), *Salix* (willow), and *Ulmus* (elm); “Weeds”—*Ambrosia* (ragweed), *Artemisia* (mugwort), and *Urtica* (nettle); and *Poaceae* (all grass).

Because admission data were available by month, we generated monthly summary measures of pollen level for each station. These were mean (reflecting the total exposure in a month) and maximum count (indicating the level of extreme exposure in the month). Data from stations that had more than 20% missing records for this period (Chester, Exeter, Cambridge, and Worcester) were excluded, leaving Ipswich, Leicester, London, Plymouth, Isle of Wight, and York. The monthly pollen statistics (mean and maximum) from the 6 stations were averaged to give monthly levels for England. (Of note, the *Alnus* count data available for March showed relatively high *Alnus*-specific counts, suggesting that our study period [April to August] did not cover the main *Alnus* season. We therefore excluded *Alnus* from further analysis.)

#### Temperature data

The monthly mean central England temperature (°C) data from 1998 to 2018 were obtained from the Met Office Hadley Centre observations datasets (Central England Temperature Series, www.metoffice.gov.uk/hadobs). This temperature time series is representative of temperature fluctuations in the Midlands region of central England, roughly in the triangular region between Bristol, Lancashire, and London. The measurements and records were maintained by the Climate Data monitoring section of the Hadley Centre, Met Office. Details of station selections and calculations has been published elsewhere.^E3^

#### Statistical models

Poisson generalized additive regression models for time series were used to examine the potentially nonlinear seasonal trend of food-related anaphylaxis admissions and its associations with temperature and pollen counts. For consistency, primary analyses used data for the period 2010 to 2018 (when admission, temperature, ***and*** pollen data were all available). In a final step, analyses that required only admissions (or admissions and temperature) data were repeated using information available from 1998 to 2018.

First, we examined the seasonal trend and the temperature-admission association. Associations between pollen level and admissions were assessed using data between April and August only (data for March and September were sparse with multiple missing data). This is the same approach used by Osborne et al^E4^ in a recent pollen-asthma study in London.

To examine seasonal trend, year (2010-2018) and month (January to December) were included as explanatory variables for total monthly admissions. To examine associations of temperature and pollen with admission, monthly temperature and monthly pollen count (for each of the 12 pollen genera/families) were added to the seasonal model separately.

All covariates, including year, month, temperature, and pollen counts, were modeled as cubic regression smooth functions s() in gam() using the mgcv() package^E5^ in R.^E6^ This approach estimates the optimal degree of freedom (*k* - 1), while allowing the user to set the maximum permissible (*k*). We increased the value of *k* until the estimated degree of freedom was stable with the minimum general cross-validation score.

RRs of admission comparing different months (highest risk vs lowest risk) were reported. As in other studies, and related to the highly skewed distribution of monthly pollen counts, RRs for pollen were reported comparing risks at the 95th and 5th percentile of exposure. The RRs for temperature were reported per 10°C change for linear association or comparing risks at extremes (95th and 5th percentile) and thresholds, if any.

To examine the possible confounding of temperature on pollen-admission associations, temperature was added to the pollen models. Interactions between pollen counts and temperature were assessed by adding an interaction term using the te() function (mgcv()).

Subgroup analysis by age group was conducted for seasonal analysis but not in the exposure-outcome analyses due to inadequate power.

Throughout all analyses, exposure in the same month and the previous month were considered. Delayed effect (lagged effect) longer than 1 month was not considered in this study because of the potentially low statistical power with the use of relatively short and low-resolution time series data (45 monthly records for pollens).
